# A Facile Methodology for Engineering the Morphology of CsPbX_3_ Perovskite Nanocrystals under Ambient Condition

**DOI:** 10.1038/srep37693

**Published:** 2016-11-25

**Authors:** Sudipta Seth, Anunay Samanta

**Affiliations:** 1 School of Chemistry, University of Hyderabad, Hyderabad 500046, India

## Abstract

A facile and highly reproducible room temperature, open atmosphere synthesis of cesium lead halide perovskite nanocrystals of six different morphologies is reported just by varying the solvent, ligand and reaction time. Sequential evolution of the quantum dots, nanoplates and nanobars in one medium and nanocubes, nanorods and nanowires in another medium is demonstrated. These perovskite nanoparticles are shown to be of excellent crystalline quality with high fluorescence quantum yield. A mechanism of the formation of nanoparticles of different shapes and sizes is proposed. Considering the key role of morphology in nanotechnology, this simple method of fabrication of a wide range of high quality nanocrystals of different shapes and sizes of all-inorganic lead halide perovskites, whose potential is already demonstrated in light emitting and photovoltaic applications, is likely to help widening the scope and utility of these materials in optoelectronic devices.

Lead halide perovskite nanocrystals (NCs) have emerged in recent years as an important class of optoelectronic materials with applications in light emitting diodes^1^ solar cells^2^ photodetectors^3^ and lasers^4^. Though the journey commenced with the hybrid perovskites, its all-inorganic counterparts (CsPbX_3_) have also drawn considerable attention in the last couple of years because of their narrow, intense and tunable emission^5^, low trap state density^6^, improved stability and charge transport properties^7^,^8^. These characteristics point to the great potential of the CsPbX_3_ NCs for future applications^6^,^9^,^10^,^11^.

As optical and electronic properties of the NCs are determined largely by their shape and size, the morphologies of the NCs are crucial to their applications in nanochemistry^12^. Though success has been achieved in other material systems^12^, only a few reports are available on the synthesis of colloidal CsPbX_3_ NCs of well-defined shape and size^5^,^13^,^14^,^15^,^16^,^17^,^18^,^19^,^20^,^21^,^22^. Highly luminescent and emission-tunable CsPbX_3_ nanocubes were reported by Kovalenko and coworkers using an air free, high temperature, hot injection approach^5^. Following a similar method, but by extending the reaction time or decreasing the temperature, luminescent nanowires^13^,^14^ and quasi-two-dimensional (2D) nanoplates (NPLs)^15^ were obtained. Nanoplatelets of controlled thickness were achieved in a room temperature approach^16^. Recently, synthesis of four different types of NCs following a room temperature reprecipitation strategy by varying the capping ligands^17^ and a similar room temperature synthesis for the preparation of nanocubes for lighting and display applications were reported^18^,^19^,^22^. Hence, the urgency of a facile, one pot synthetic approach to tune the morphology (isotropic and anisotropic) of colloidal CsPbX_3_ (X = Cl, Br, I) NCs is the motivation behind our work.

Here, we report a room temperature, open atmosphere anti-solvent precipitation method for the synthesis of six different colloidal CsPbX_3_ NCs just by varying the medium, ligand concentration and reaction time. In ethyl acetate, zero-dimensional (0D) quasi-cubic quantum dots (QDs), 2D NPLs and nanobars are synthesized in sequential manner using oleylamine (OLA) and oleic acid (OA) as capping ligand. In toluene, nanocubes of different sizes were obtained at higher concentration of OLA, whereas at lower concentration, first the nanocubes, then the nanorods of increasing length and finally, nanowires are obtained. All these NCs exhibit shape-dependent photoluminescence (PL) behavior. To the best of our knowledge, this is the first room temperature and open atmosphere synthesis of size-controllable CsPbX_3_ nanocubes, nanobars and nanowires with nanorod of controllable aspect ratio as intermediate.

## Result and Discussion

The NCs are fabricated by preparing a 1:1 molar precursor solution of PbBr_2_ and CsBr in polar dimethyl formamide (DMF, 4 mL) and then adding this solution (200 μL) drop wise to a vigorously stirred reaction media containing definite quantities of OLA (20–70 μL) and OA (0.2–0.5 mL) in a larger amount (4 mL) of a less polar solvent (anti-solvent) at room temperature. The morphologies were precisely controlled by varying the anti-solvent (ethyl acetate and toluene), quantity of the capping agent (OLA and OA) and reaction time, the details of which are provided in tabular form as supporting information (Table S1–3). In ethyl acetate, we obtained the QDs, NPLs and nanobars and in toluene, nanocubes of different sizes, nanorods of increasing length and nanowires are obtained.

### Quantum Dots

**Figure 1:**
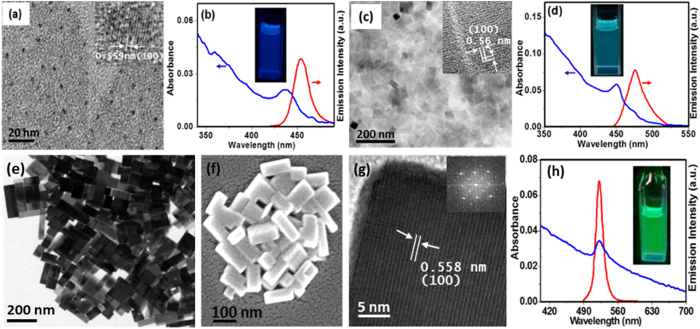
CsPbBr_3_ nanocrystals in ethyl acetate. (**a**) TEM image of the QDs and HR-TEM image (inset) of a single QD, (**b**) optical absorption and PL (blue) spectra of the QDs, (**c**) TEM and HR-TEM (inset) images of the NPLs, (**d**) optical absorption and PL (cyan) spectra of the NPLs, (**e**–**g**) TEM, FESEM and HR-TEM (inset shows FFT) images, respectively, of the nanobars and (**h**) optical absorption and PL (green) spectra of the nanobars.

When ethyl acetate is used as the anti-solvent, 2.6 ± 0.9 nm sized quasi-cubic CsPbBr_3_ QDs are formed immediately on addition of the precursor solution. Figure 1a and Figure S1 (SI) show the transmission electron microscopy (TEM) and high-resolution transmission electron microscopy (HR-TEM) images from which we obtain an inter-planar spacing of 0.559 nm corresponding to the (100) plane. Cubic crystal phase of these QDs is determined from the powder X-ray diffraction (PXRD) study (Figure S1, SI). The bluish-green colored QD solution shows an intense blue PL (quantum yield (QY) 27%, Table S4, SI) with first excitonic and PL emission peak at around 437 and 454 nm, respectively (Fig. 1b). Time-resolved PL behavior is best described by three exponential decay components (Figure S1, SI), giving an average PL lifetime of 3.9 ns.

### Nanoplates

Allowing the reaction up to 10 minutes, a few unit cell thick, square shaped CsPbBr_3_ NPLs of 60 nm edge length are obtained. TEM, HR-TEM and field emissive scanning electron microscopy (FESEM) images are shown in Fig. 1c and Figure S2 (SI) respectively. HR-TEM images show that the single crystalline NPLs are bound by the (100) planes. The double peaks at around 30° in the PXRD pattern (Figure S2, SI) confirm the orthorhombic phase^13^ and the energy dispersive X-ray (EDX) spectroscopy measurements (Figure S2, SI) confirm 1:1:3 ratio of the Cs, Pb and Br atoms in synthesized CsPbBr_3_ NPLs. Typical thickness of the obtained NPLs are ~4.8 nm, as evident from the atomic force microscopy images (Figure S2, SI) and that the thickness represents that of the primary NPLs capped with the organic ligands^15^,^16^. Cyan emitting NPLs exhibit the first excitonic and PL emission peak at 450 and 476 nm (Fig. 1d) with PL QY of 19%. Triexponential fitting of the PL decay profile yields an average lifetime of 5.1 ns (Figure S2 and Table S4, SI).

### Nanobars

With further increase in reaction time the nanobars start forming at the expense of the NPLs. After 40 hours, the reaction medium contains mostly the nanobars with an average aspect ratio of 2.55 (~140 nm long and ~55 nm wide). Figure 1 shows the TEM and FESEM images of the nanobars with very low size dispersion. That the nanobars are highly crystalline in nature is evident from the HR-TEM images (Fig. 1 and S3, SI), their corresponding FFTs (inset) and selected area electron diffraction (SAED) pattern (Figure S3, SI). Additional images are shown in SI. Single crystalline nanobars are of orthorhombic phase (Figure S3, SI) and bound mainly by (100) and (110) in the in-plane and side-plane facets. EDX spectrum (Figure S3, SI) confirms the 1:1:3 ratio of the constituent elements. Both, first absorption onset and emission peak of the nanobars appear at 522 nm (Fig. 1h). Unlike the other morphologies, the PL QY of the nanobars is much higher (61%) and the PL decay profile is characterized by a biexponential kinetics (Figure S3, SI) with an improvement in individual lifetime components (Table S4, SI) and increase in the average lifetime (14.2 ns). The high PL QY and long PL lifetime of the nanobars indicate less charge trapping sites as compared to the other NCs and majorly excitonic radiative recombination. Superior quality of the synthesized nanobars was further confirmed by exposing the NPLs and nanobars under electron beam. While the NPLs degrade within 10 seconds, the nanobars remain unaffected even after 10 minutes of exposure (Figure S4, SI).

### Nanocubes of Different Sizes

**Figure 2:**
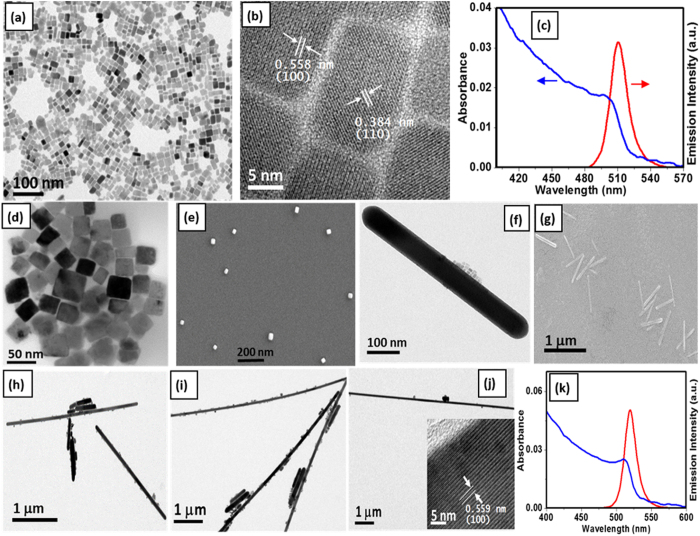
CsPbBr_3_ nanocrystals in toluene. (**a**) TEM, (**b**) HR-TEM image and (**c**) optical absorption and PL spectra of nanocubes (~12 nm), (**d**) TEM and (**e**) FESEM images of larger nanocubes (~34 nm), (**f**) TEM and (**g**) FESEM images of nanorods, (**h**,**i** and **j**) TEM images of increasing length of the nanorods after 5, 25 and 40 hours of reaction respectively, (**j**-Inset) HR-TEM image and (**k**) absorption and PL spectra of the corresponding nanowires.

By changing the anti-solvent to toluene and keeping OLA (20–70 μl) and OA (200 μl) as capping ligand, we can further manipulate the morphology of CsPbBr_3_ NCs. Well-crystallized nanocubes of 12 ± 2 nm size are obtained at the initial stage of the reaction following a similar procedure as before. TEM and HR-TEM images are shown in Fig. 2a,b and S5 (SI). PXRD and EDX spectra (Figure S5, SI) confirm the formation of CsPbBr_3_ nanocubes. First absorption onset and emission peak appear at 497 and 510 nm respectively (Fig. 2c). PL time profile of this material shows a triexponential decay behavior with an average lifetime of 11.3 ns. High PL QY of ~77% and good crystalline quality suggest that the nanocubes are largely free from charge trapping mid band gap trap states indicating possible application in optoelectronic devices.

Extension of the reaction time up to 1 hour leads to the formation of another two morphologies for two different OLA concentrations. When 70 μl OLA is used, larger nanocubes of ~34 nm edge-length are formed as evident from the TEM (Fig. 2d, S6, SI) and FESEM (Fig. 2e) images. Well crystalline nanocubes retain the cubic phase and bound by (100) and (110) planes (Figure S6, SI). Red-shifted first absorption onset and emission peak compared to smaller nanocubes (~12 nm) appear at 502 and 514 nm (Figure S6, SI), respectively. PL QY and lifetime of the nanocubes are 54% and 13.5 ns (Figure S6, SI), respectively. Further increase in reaction time up to 50 hours or more ends up with larger nanocubes and few nanorods of larger dimension (Figure S7, SI).

### Nanorods and Nanowires

Less quantity of OLA (20 μl) provides a faster kinetics and forms CsPbBr_3_ nanorods of good crystalline quality (after 1 hour) at the expense of nanocubes formed initially. TEM (Fig. 2f and S8, SI) and FESEM (Fig. 2g) images show an average nanorod length and diameter of 800 and 70 nm, respectively. Single crystalline nanorods grow along <100> direction as evident from the HR-TEM image (Figure S8, SI) and corresponding FFT (inset). These nanorods are of orthorhombic phase (Figure S8, SI) and show PL QY of 34%. First absorption onset and emission peak appear at 505 and 516 nm (Figure S8, SI). Triexponential fitting of the PL decay profile gives an average lifetime of 8.9 ns (Figure S8, SI). With further increase in reaction time, the length of the nanorods increases and density of the nanocubes decreases. As can be seen from Fig. 2h–j and Table S2 (SI), the aspect ratio of the nanorods increases with time and finally, after 40 hours of reaction, nanowires of diameter ~70 nm and length ≥15 μm are obtained. As evident from HR-TEM (Fig. 2j inset, S9, SI) and SAED pattern (Figure S3, SI) these nanowires are of very good crystalline quality with <100> growth direction. Nanowires are of orthorhombic phase and the ratio of Cs, Pb, Br to be close to 1:1:3. First absorption onset and emission peak appear at 511 and 520 nm, respectively. These nanowires have an average PL lifetime of 11.4 ns and QY of 29%. Both nanorods and nanowires obtained by the present method are of better crystalline quality and show improved PL QY as compared to the other reports^13^,^17^.

X-ray photoelectron spectroscopy (XPS) measurements have been performed on different CsPbBr_3_ morphologies to further investigate the quality and composition. The survey X-ray photo electron spectra (Figure S10) show the peaks corresponding to Cs 3d, Pb 4f, Br 3d. XPS peak areas of the survey curve provide a ratio of 1:1:3 of Cs, Pb and Br of the NCs capped with oleic acid and oleylamine. The as obtained results are consistent with the literature^16^ and confirm the identity and purity of our samples.

### Effect of Other Solvents

The results obtained by following a similar synthetic protocol but using other organic solvents of different polarity as anti-solvent are summarized in Table S3 (SI). In chloroform, smaller NCs emitting at 487 nm and nanocubes emitting at 510 nm (~12 nm) are formed at the early stages of the reaction. With increase in time, the nanocubes become larger in dimension and undergo slow degradation showing a decrease in PL after 24 hours of reaction (Figure S11, SI). It is found that polarity of the used anti-solvent is crucial for the synthesis of the NC and its stability. The NCs degrade readily when more polar anti-solvents are used (Table S3, SI). Notably, during the reaction in a given anti-solvent, the morphologies evolved in a sequential manner and hence, different intermediates can coexist at a time (Table S1–3; SI). Different NC population was separated from each other by the use of size-selective precipitation method.

### Anion exchange

**Figure 3:**
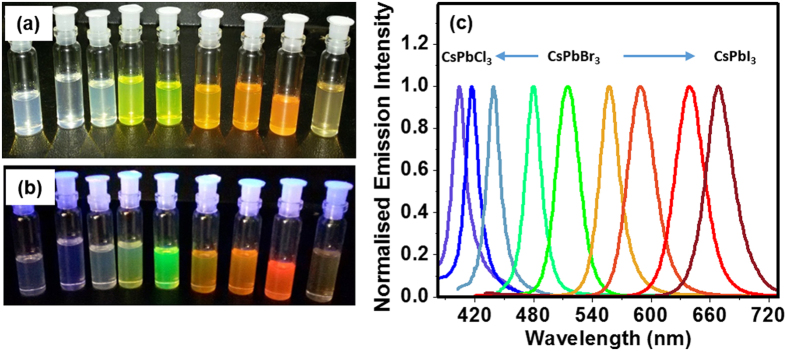
Series of CsPbX_3_ solutions collected at different stages of the anion exchange of CsPbBr_3_ nanocubes (~34 nm) with PbCl_2_ and PbI_2_ in toluene at room temperature and open atmosphere under day light (**a**) and ultraviolet light (**b**), and their PL spectra (**c**).

CsPbX_3_ (X = Cl, I) NCs are prepared at room temperature using simple and fast anion exchange method^23^,^24^ by adding definite proportion of PbCl_2_ or PbI_2_ to the CsPbBr_3_ NC solution. Figure 3 illustrates that the entire visible spectral window (405–700 nm) can be covered by the anion exchange process on ~34 nm sized nanocubes in toluene. The method is found to be applicable for all other morphologies.

### Formation Mechanism

The nature of the ligand, its quantity, solvent and reaction time are found to play important role in determining the shape, size and luminescence properties of the NCs. Recognizing that OLA binds more strongly to the NCs compared to OA and considering the observations that (i) 100% OA leads to the formation of nonfluorescent bulk NCs with a wide size distribution (50–500 nm) and no particle formation takes place in its absence (Table S1, 2, SI), (ii) increasing OLA concentration slows down the formation rate of the NCs and excess of it retards the nucleation process completely (Table S1, 2, SI), and (iii) different morphologies can be observed only in the presence of both OA and OLA, we postulate the following mechanism of formation of different morphologies.

**Figure 4:**
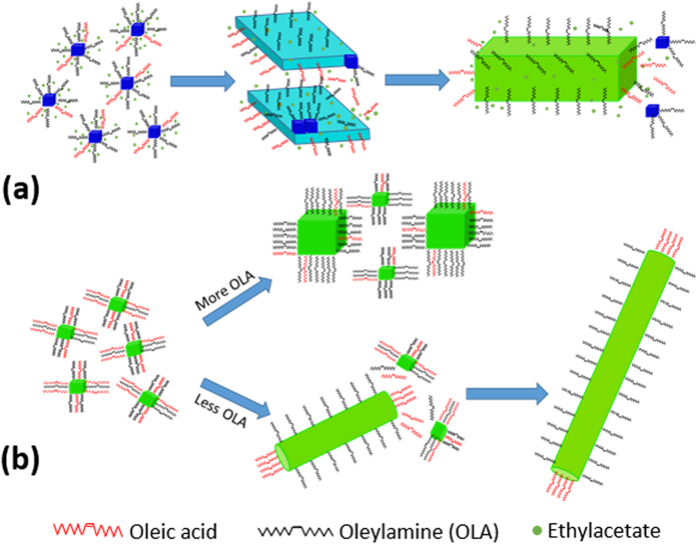
Mechanism proposed for the formation of (**a**) NPLs (cyan) and nanobars (green) from blue-emitting QDs in ethyl acetate and (**b**) larger nanocubes, nanorods and nanowires from smaller nanocubes in toluene.

In less polar solvents, toluene and ethyl acetate, long alkyl chain hydrophobic ligands form micelle of specific sizes, which lead to the formation of small NCs. Quasi-cubic QDs in ethyl acetate and cubic NCs in toluene are formed inside the micelle because of inherent cubicity of CsPbBr_3_ perovskite material. In ethyl acetate, 2D growth along a plane leading to the formation of NPLs can only be explained if OLA, the stronger ligand of the two, binds preferentially to a given plane. As ethyl acetate is more polar than toluene, it acts both as a solvent and a nucleophile and can remove some OLA from the bound surface of the NPLs where small QDs can attach easily. This process can lead to an anisotropic growth of the NPLs with time and result in the formation of the nanobars as final product (Fig. 4). In toluene, in the presence of a small amount of OLA nanoparticles start growing along a direction along which less OLA (hence, more OA) is present. Nanocubes break their inherent symmetry and add up in a unidirectional manner to form nanorod and finally nanowires at longer time, as illustrated in Fig. 4. In the presence of a larger quantity of OLA, the nanocube surfaces become more protected in all directions and consequently, larger nanocubes (oligomers) are formed due to self-aggregation at longer reaction time (Fig. 4).

## Conclusion

In conclusion, several CsPbX_3_ NCs of different shapes and sizes (QDs, NPLs, nanobars, small and large nanocubes, nanorods and nanowires) are obtained following an anti-solvent precipitation method under ambient condition. These NCs are of excellent crystalline quality and show size, shape and composition dependent PL properties. High quality nanobar is shown to be a new member of the CsPbX_3_ perovskite NC family. This simple method of fabrication of a broad range of NCs of various shapes and sizes is likely to boost the potential of this class of promising materials in light emitting and photovoltaic applications. Study of photo-induced charge separation and recombination dynamics involving these materials is currently underway.

## Experimental details

### Chemicals

CsBr (99.999%, Aldrich), PbCl_2_ (99.999%, Aldrich), PbBr_2_ (≥98%, Aldrich), PbI_2_ (99.999%, Aldrich), oleylamine (OLA, 70%, Aldrich), oleic acid (OA, 90%, Aldrich), dimethyl sulfoxide (DMSO, 99.9%, Aldrich), dimethyl formamide (DMF, 99%, Aldrich), ethyl acetate (AR, Merck), toluene (AR, Finar), hexane (GR, Merck), acetone (GR, Fisher scientific) and 1-butanol (GR, Merck) were used as received.

### Preparation of the precursor solution

In a 20 mL reagent bottle 0.15 mmol CsBr and 0.15 mmol PbBr_2_ was dissolved in 4 mL of DMF.

### Synthesis of CsPbBr_3_ nanocrystals (NCs)

In a 50 mL round bottom flask 4 mL anti-solvent (less polar solvents) was loaded with desired amount of OLA and OA as the capping ligand and kept in a vigorously stirring condition. 200 μL of precursor solution was added drop wise into the stirring solution. Depending on the anti-solvent used and amount of ligands added, color of the resultant solution changes from bluish green to green to yellow (in ethyl acetate) or from greenish yellow to yellow (in most of the other organic solvents) with increase in time. The conditions maintained for obtaining specific size and shape of the NCs are summarized in Table S1–3.

### Separation and purification of the NCs

The crude solutions collected at different stages of the reaction were centrifuged at 5000 rpm for 6 minutes. When a mixture of the NCs was present, centrifugation was done for a longer period at 7500 rpm. Following the centrifugation, the supernatant liquid was discarded, the precipitate was washed with ethyl acetate and then dispersed in toluene or hexane.

### Anion exchange process

0.15 mmol PbX_2_ (X = Cl, I) was dissolved in 5 mL of DMF and added drop wise to the stirring CsPbBr_3_ NC solution. By controlling the amount of added PbX_2_ solution we can easily tune the emission properties of these NCs. The reaction reaches to equilibrium after allowing it for some time then the NCs were separated by centrifugation for further studies. Anion exchange technique is very facile at ambient condition and was successfully applied for all NCs obtained in this work.

### Instrumentation and methods

Tecnai G2 FE1 F12 transmission Electron microscope (TEM) at an accelerating voltage of 200 kV was used to obtain images, high resolution images and selected area electron diffraction (SAED) pattern. Field-emission scanning electron microscope (FE-SEM) imaging was carried out using a Carl Zeiss model Ultra 55 microscope. Atomic force microscope (AFM) images were recorded on a NT-MDT Model solver Pro-M AFM in a semi-contact mode using a tip having a force constant of 12 nm^−1^. Powder X-ray diffraction (PXRD) of the NCs were recorded on a SMART Bruker D8 Advance X-ray Diffractometer using Cu-Kα radiation (λ = 1.5406 A°). X-ray photoelectron spectra (XPS) of the samples were recorded with a custom built ambient pressure photoelectron spectrometer (APPES) (Prevac, Poland) equipped with a VG Scienta’s R3000HP analyzer and MX650 monochromator. Monochromatic Al Ka X-rays were generated at 200 W and used for measuring the X-ray photoelectron spectrum (XPS) of the samples. Base pressure in the analysis chamber was maintained in the range of 5 × 10^−10^ Torr. The energy resolution of the spectrometer was set at 0.7 eV at a pass energy of 50 eV. Binding energy (BE) was calibrated with respect to Au 4f7/2 core level at 84.0 eV. Samples were flooded with low energy electrons for efficient charge neutralisation. Steady state absorption and photoluminescence (PL) spectra were taken in an UV-vis spectrophotometer (Cary 100, Varian) and fluorescence spectrometer (Fluorolog 3, Horiba Jobin Yvon). PL quantum yield (QY) of different NCs was calculated using Coumarin 153 as reference (QY = 0.56 in acetonitrile). For PL lifetime measurements, Horiba Jobin Yvon IBH TCSPC spectrometer was used. A Nano LED laser source of output at 405 nm (fwhm: 200 ps) at 1 MHz repetition rate was used as the excitation source. PL decay curves were fitted to a multi-exponential decay function of the form,


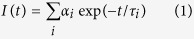


where, τ_i_ are the lifetime components and α_i_ are the corresponding exponents. The average lifetime, <τ>, reported in this work is defined as,


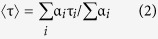


## Additional Information

**How to cite this article**: Seth, S. and Samanta, A. A Facile Methodology for Engineering the Morphology of CsPbX_3_ Perovskite Nanocrystals under Ambient Condition. *Sci. Rep.* **6**, 37693; doi: 10.1038/srep37693 (2016).

**Publisher's note:** Springer Nature remains neutral with regard to jurisdictional claims in published maps and institutional affiliations.

## Supplementary Material

Supplementary Information
