# Supplementary material for: A Facile Methodology for Engineering the Morphology of CsPbX3 Perovskite Nanocrystals under Ambient Condition
Source: Sci Rep. 2016 Nov 25;6:37693. doi: 10.1038/srep37693 (PMC5122874; doi:10.1038/srep37693)
Supplement: Supplementary Information [file srep37693-s1.pdf]

Supporting Information

A Facile Methodology for Engineering the Morphology of $CsPbX_3$ Perovskite Nanocrystals under Ambient Condition

Sudipta Seth and Anunay Samanta*

School of Chemistry, University of Hyderabad, Hyderabad 500046, India

*Corresponding author, E-mail: anunay@uohyd.ac.in

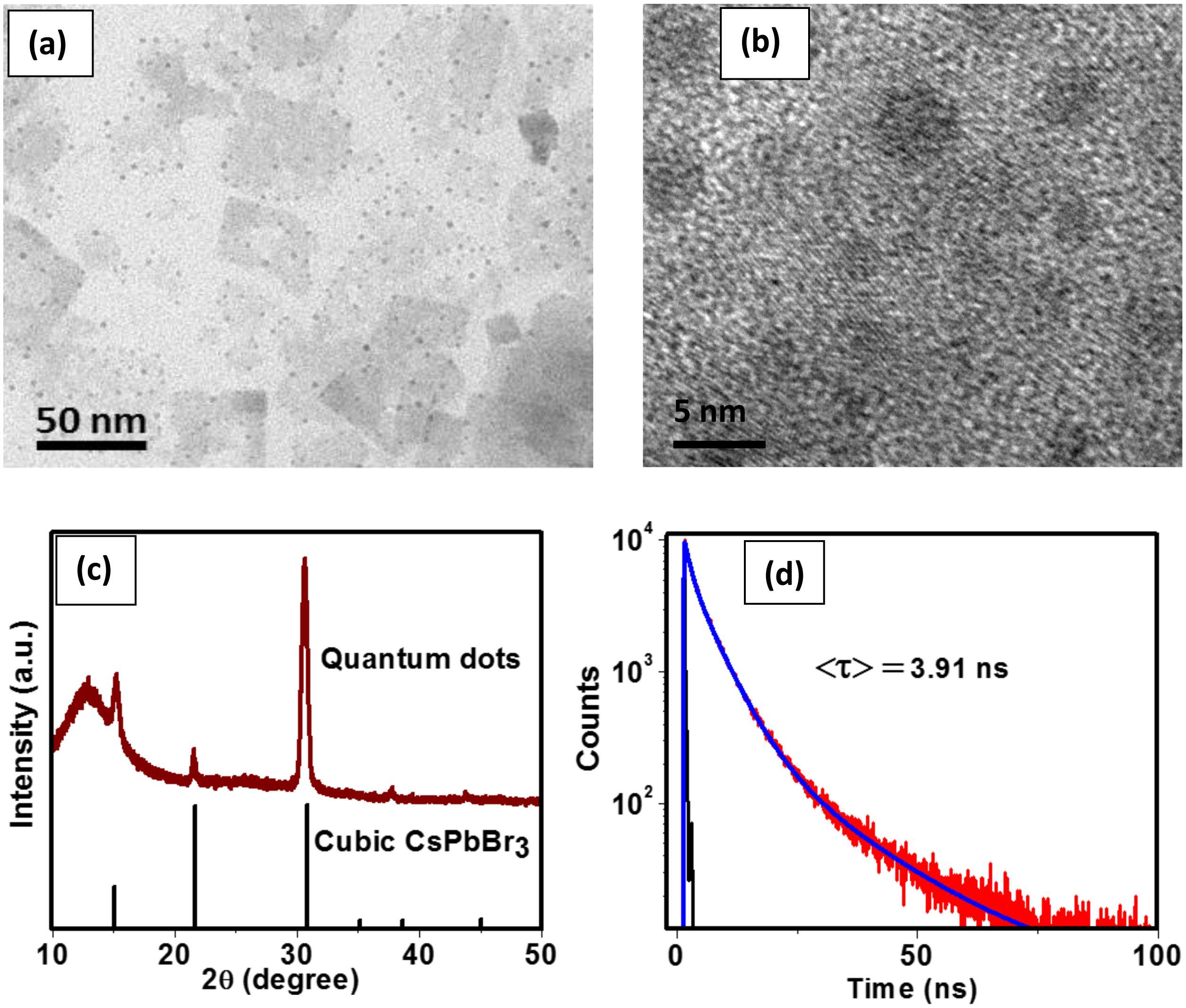

Figure S1. $CsPbBr_3$ quasi-cubic quantum dots formed in ethyl acetate within 1 min of the reaction. (a) TEM, (b) HR-TEM images, (c) PXRD pattern of the QDs with standard diffraction pattern of the cubic phase obtained from PCPDFWIN #75-0412. Below 15 degree the broad peak is because of instrument artifacts (see Figure S15 for confirmation). (d) Time-resolved PL decay and fitting with a tri-exponential function, excitation wavelength = 405 nm.

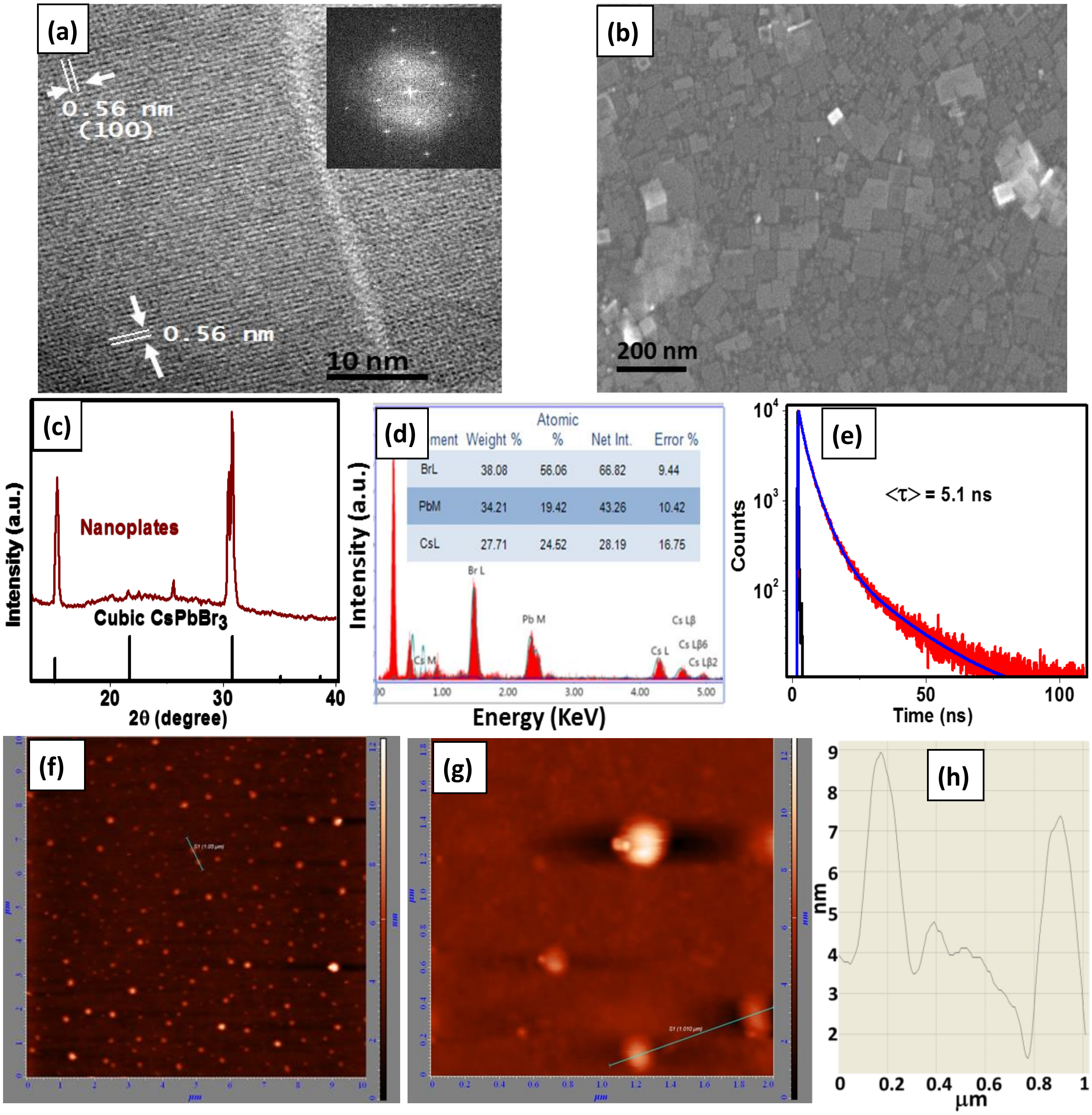

Figure S2. $CsPbBr_3$ nanoplates (NPLs) formed in ethyl acetate after 10 minutes of the reaction. (a) HR-TEM, (b) FESEM images, (c) PXRD pattern of the NPLs with standard diffraction pattern of the cubic phase obtained from PCPDFWIN #75-0412. Below 15 degree the broad peak is because of instrument artifacts. (d) EDX spectra and atomic composition of the constituent elements, (e) time-resolved PL decay and its fitting with a triexponential function. Excitation wavelength = 405 nm. (f, g, h) AFM images show thickness of the NPLs around 4.8 nm.

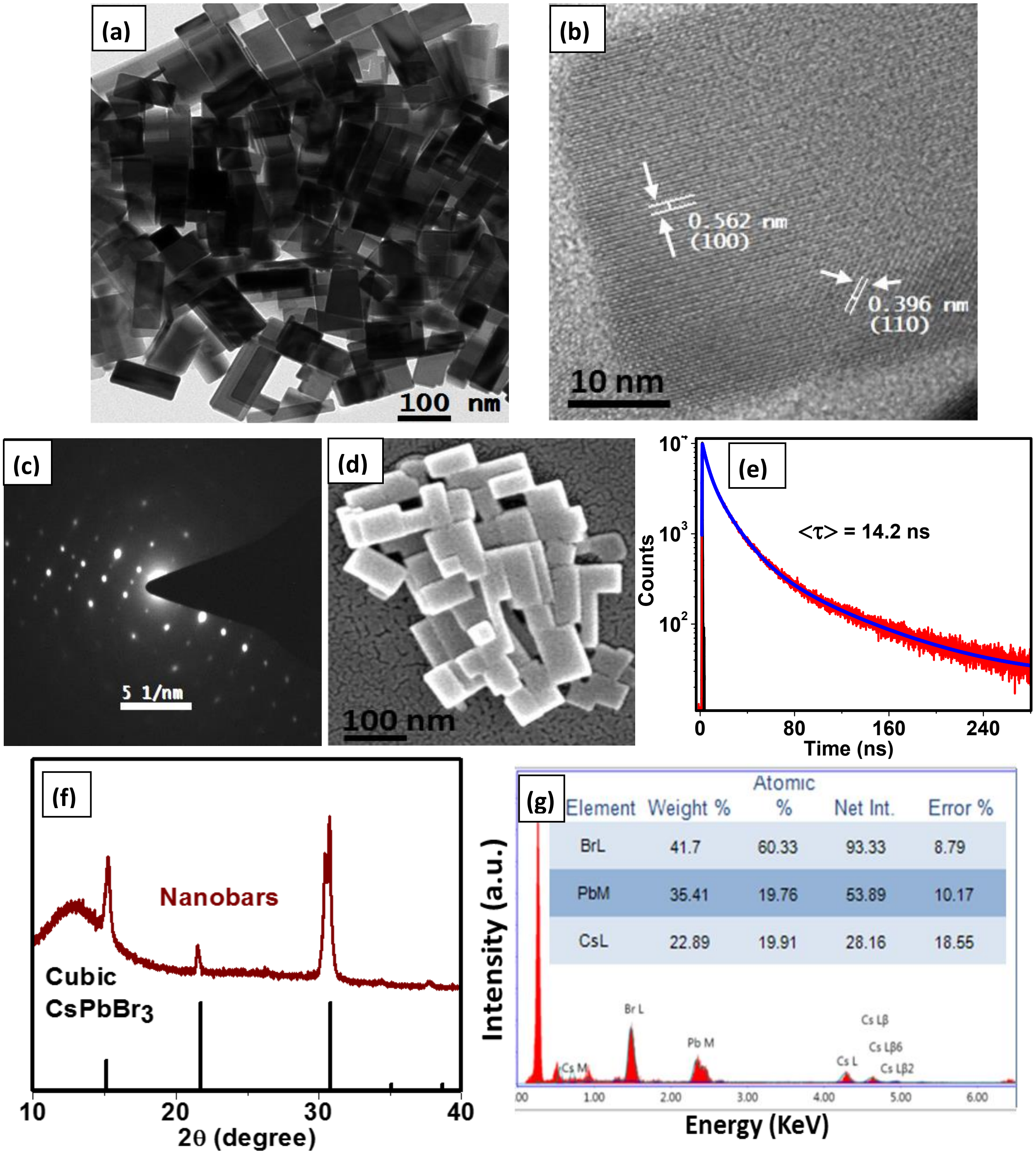

Figure S3. $CsPbBr_3$ nanobars formed in ethyl acetate after 40 hours of the reaction. (a) TEM, (b) HR-TEM images, (c) SAED pattern, (d) FESEM images of the $CsPbBr_3$ nanobars (e) time-resolved photoluminescence decay and fitting with a bi-exponential function, excitation wavelength = 405 nm. (f) PXRD pattern of the nanobars with standard diffraction pattern for cubes obtained from PCPDFWIN #75-0412. Below 15 degree the broad peak is because of instrument artifacts. (g) EDX spectrum and atomic composition of the nanobars.

(a)

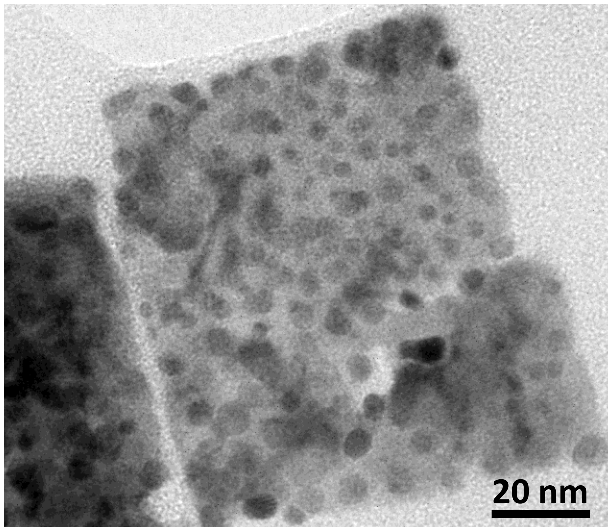

(b)

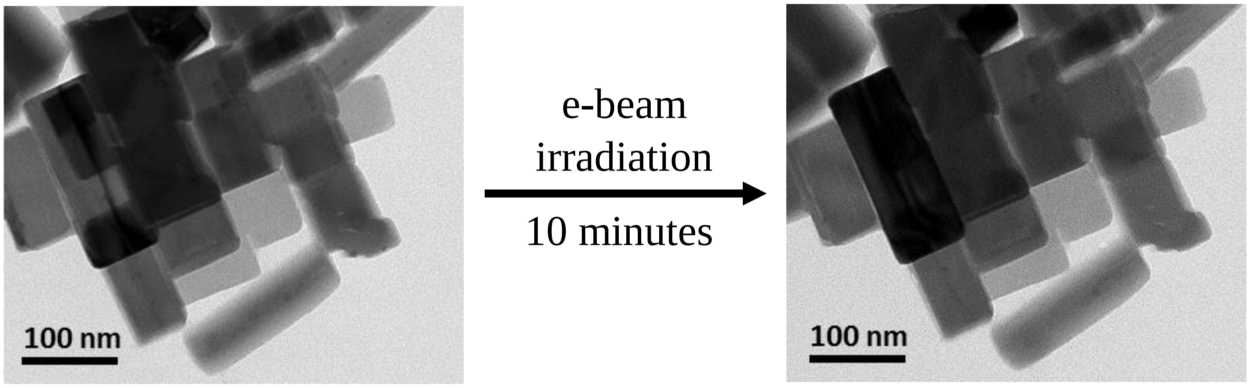

Figure S4. TEM images of $CsPbBr_3$ (a) nanoplates and (b) nanobars after electron beam irradiation for 10 seconds and 10 minutes respectively.

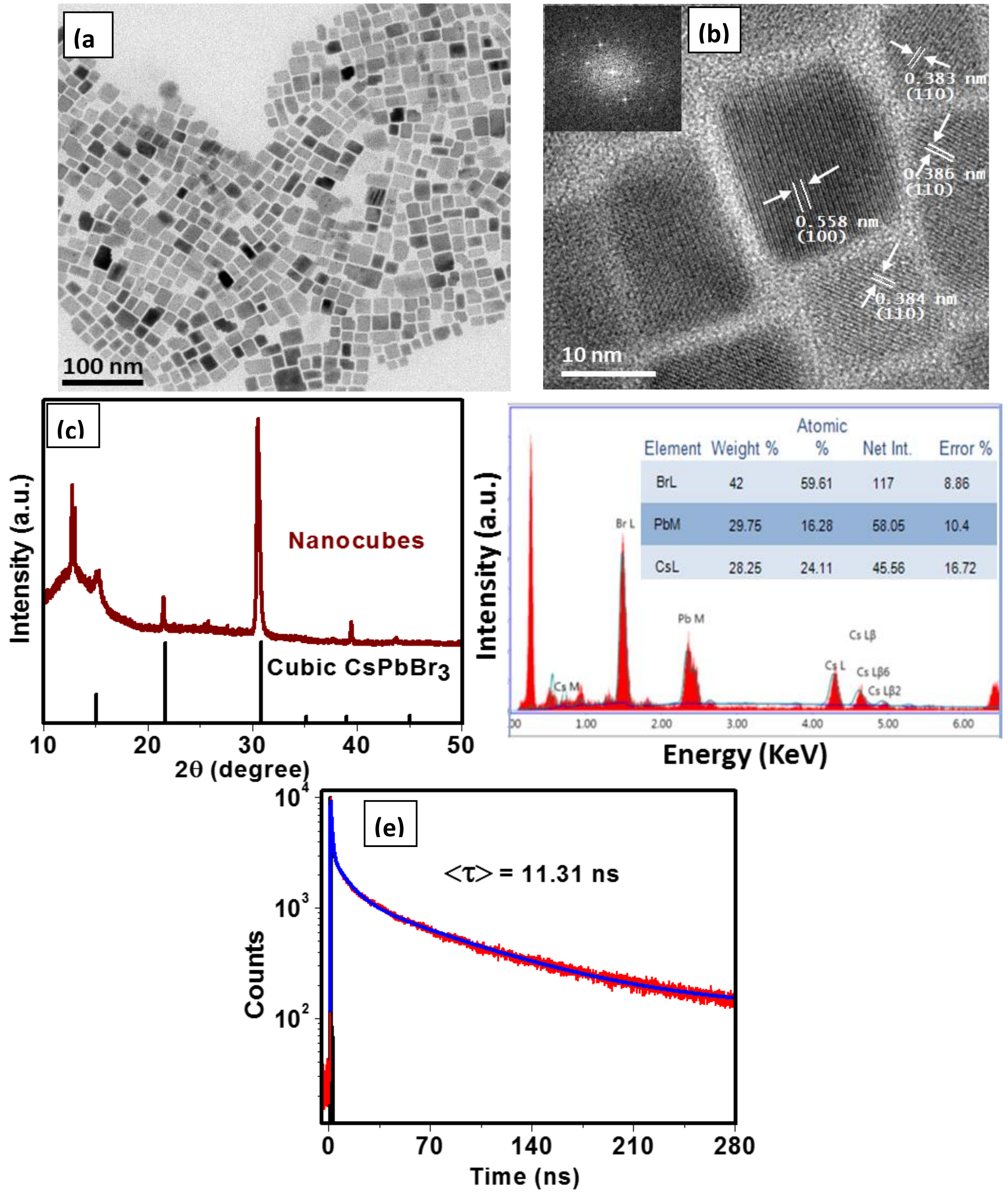

Figure S5. $CsPbBr_3$ nanocubes (~12 nm) formed in toluene after 1 min of the reaction. (a) TEM, (b) HR-TEM (inset: corresponding FFT of the central cube) images, (c) PXRD pattern of the nanocubes along with standard cubic phase diffraction pattern obtained from PCPDFWIN #75-0412. Below 15 degree the broad peak is because of instrument artifacts. (d) EDX spectrum shows close to 1:1:3 proportion of the constituent elements in the nanocubes. (e) Time-resolved photoluminescence decay and fitting with a tri-exponential function, excitation wavelength = 405 nm.

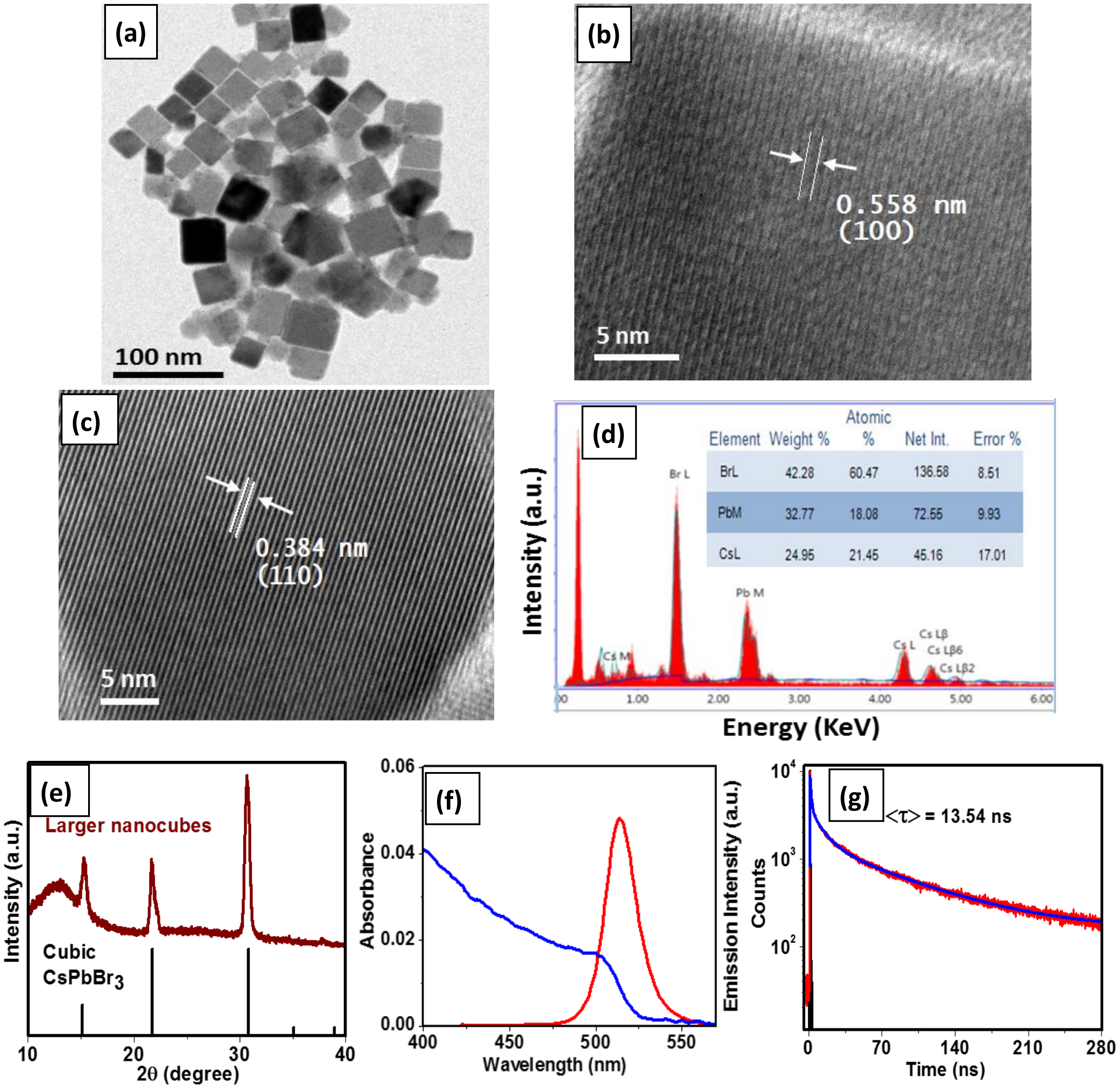

Figure S6. Larger $CsPbBr_3$ nanocubes formed in toluene after 1 hour of the reaction when 70 µl OLA was added. (a) TEM, (b, c) HR-TEM images, (d) EDX spectrum and atomic composition of the nanocubes (e) PXRD pattern of the nanocubes along with standard diffraction pattern obtained from PCPDFWIN #75-0412. Below 15 degree the broad peak is because of instrument artifacts. (f) Absorption and PL spectra, (g) time-resolved photoluminescence decay and fitting with a tri-exponential function, excitation wavelength = 405 nm.

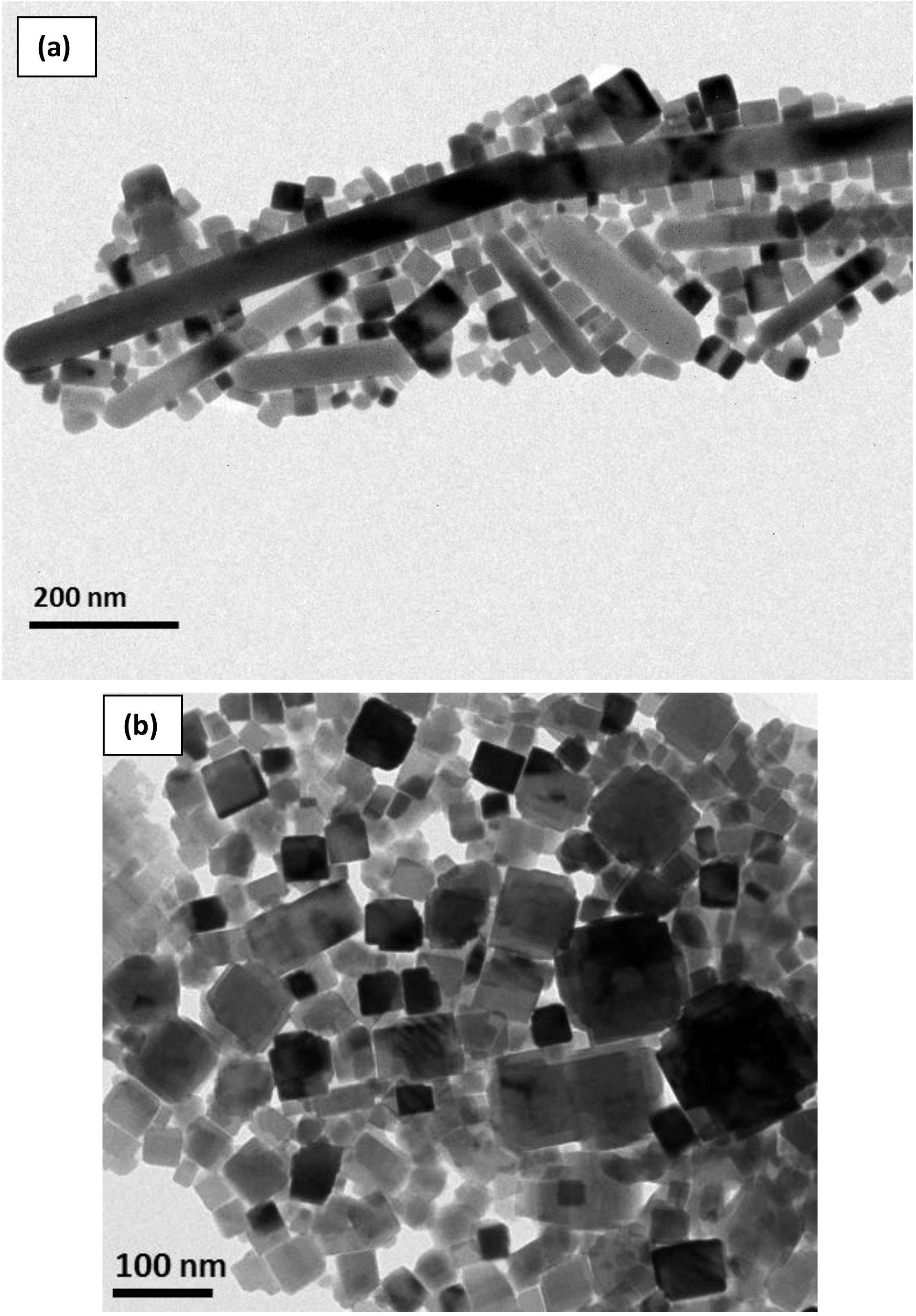

Figure S7. (a), (b) TEM images $CsPbBr_3$ nanocubes and nanorods of larger dimension formed in toluene after 50 hours of the reaction when 70 μl OLA was used. Images are taken from different places of the same sample.

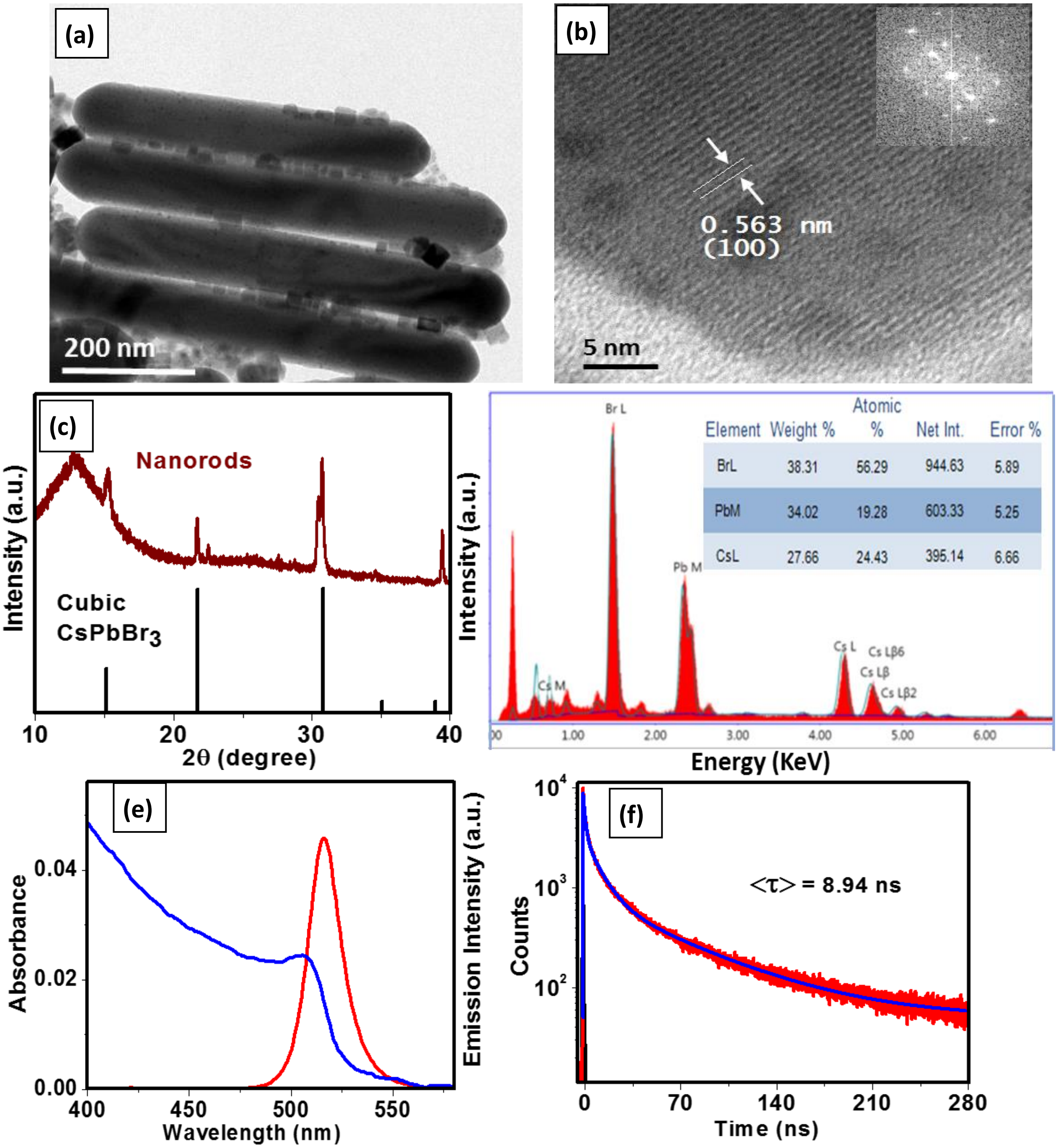

Figure S8. $CsPbBr_3$ nanorods formed in toluene after 1 hour of the reaction when 20 µl OLA was used. (a) TEM, (b) HR-TEM (inset: corresponding FFT) images of the nanorods. (c) PXRD pattern of the nanorods shows an orthorhombic phase as evident from the double peaks at ~30°. Below 15 degree the broad peak is because of instrument artifacts. (d) EDX spectrum shows 1:1:3 atomic composition in the nanorods. (e) Absorption and PL spectra and (f) time-resolved photoluminescence decay and fitting with a tri-exponential function, excitation wavelength = 405 nm.

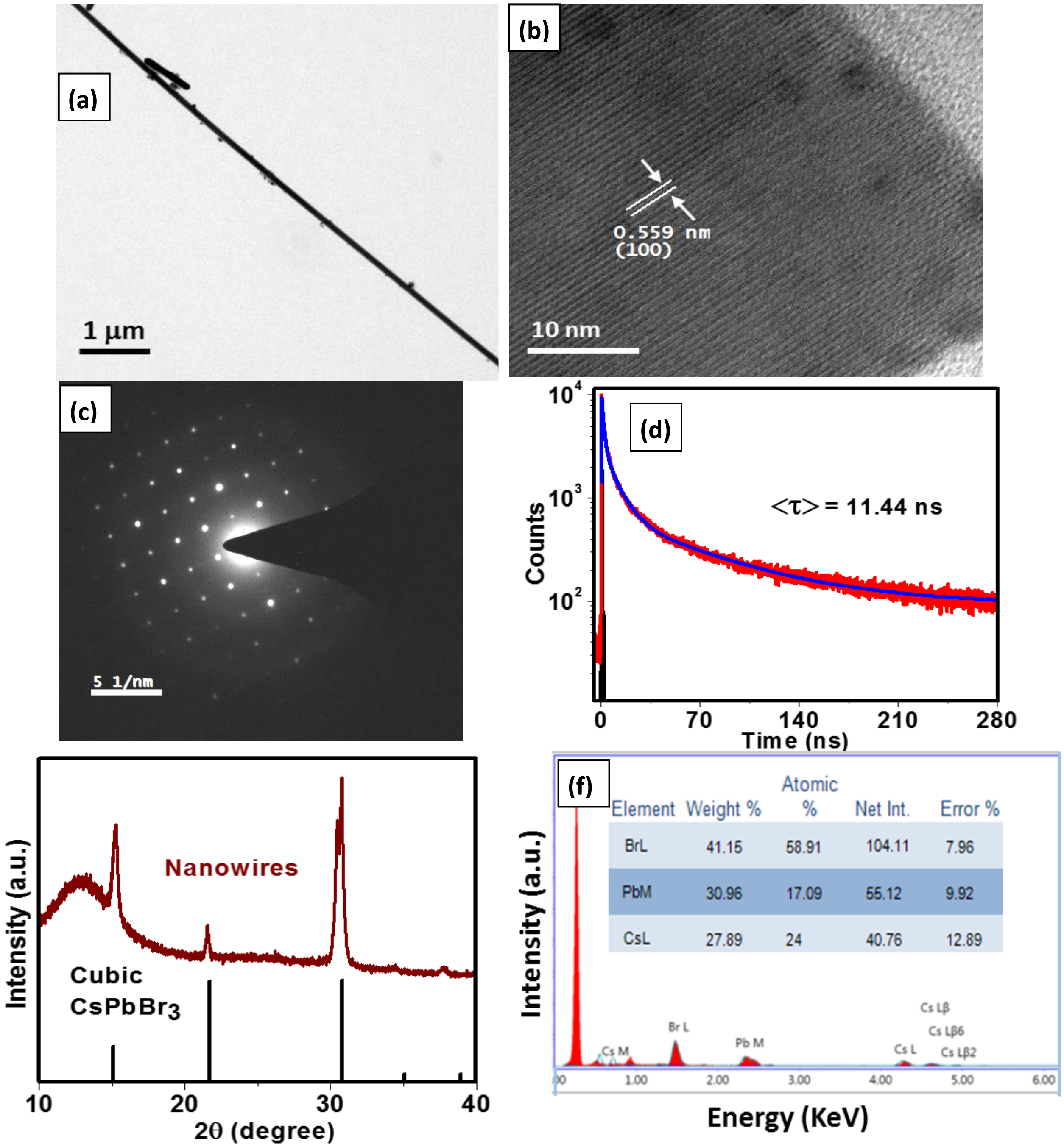

Figure S9. $CsPbBr_3$ nanowires formed in toluene after 40 hour of the reaction when 20 μl OLA was added. (a) TEM, (b) HR-TEM images, (c) SAED pattern of the nanowires. (d) Time-resolved photoluminescence decay and fitting with a tri-exponential function, excitation wavelength = 405 nm. (e) PXRD pattern of the nanowires compared with standard diffraction pattern of the cubes obtained from PCPDFWIN #75-0412. Below 15 degree the broad peak is because of instrument artifacts. (f) EDX spectrum shows the atomic composition of the nanowires close to 1:1:3.

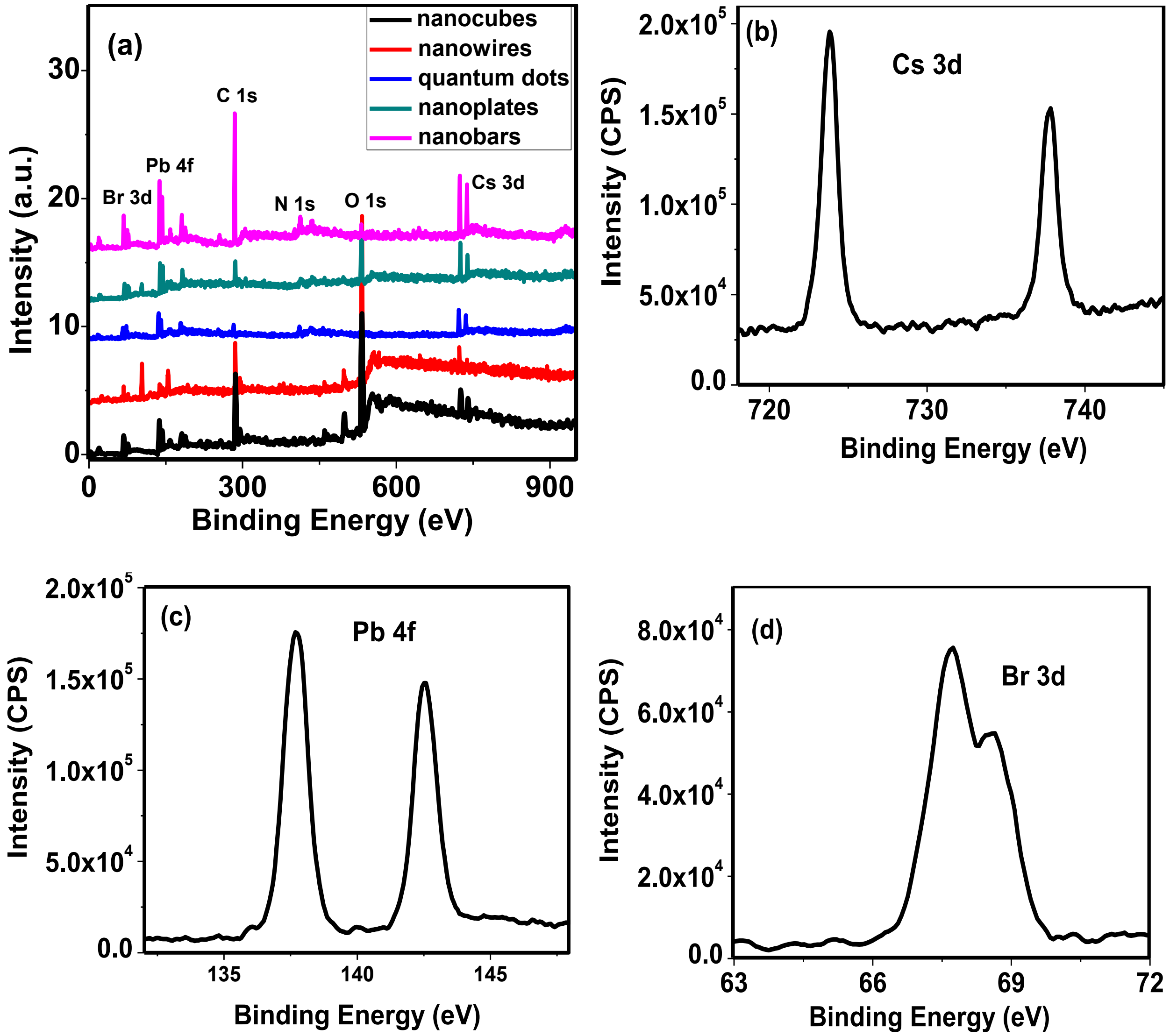

Figure S10. XPS spectra of different morphologies CsPbBr3 nanocrystal. Survey XPS with defined peaks at different binding energies (a) and high-resolution XPS of (b) Cs 3d, (c) Pb 4f, (d) Br 3d of CsPbBr3 quantum dots.

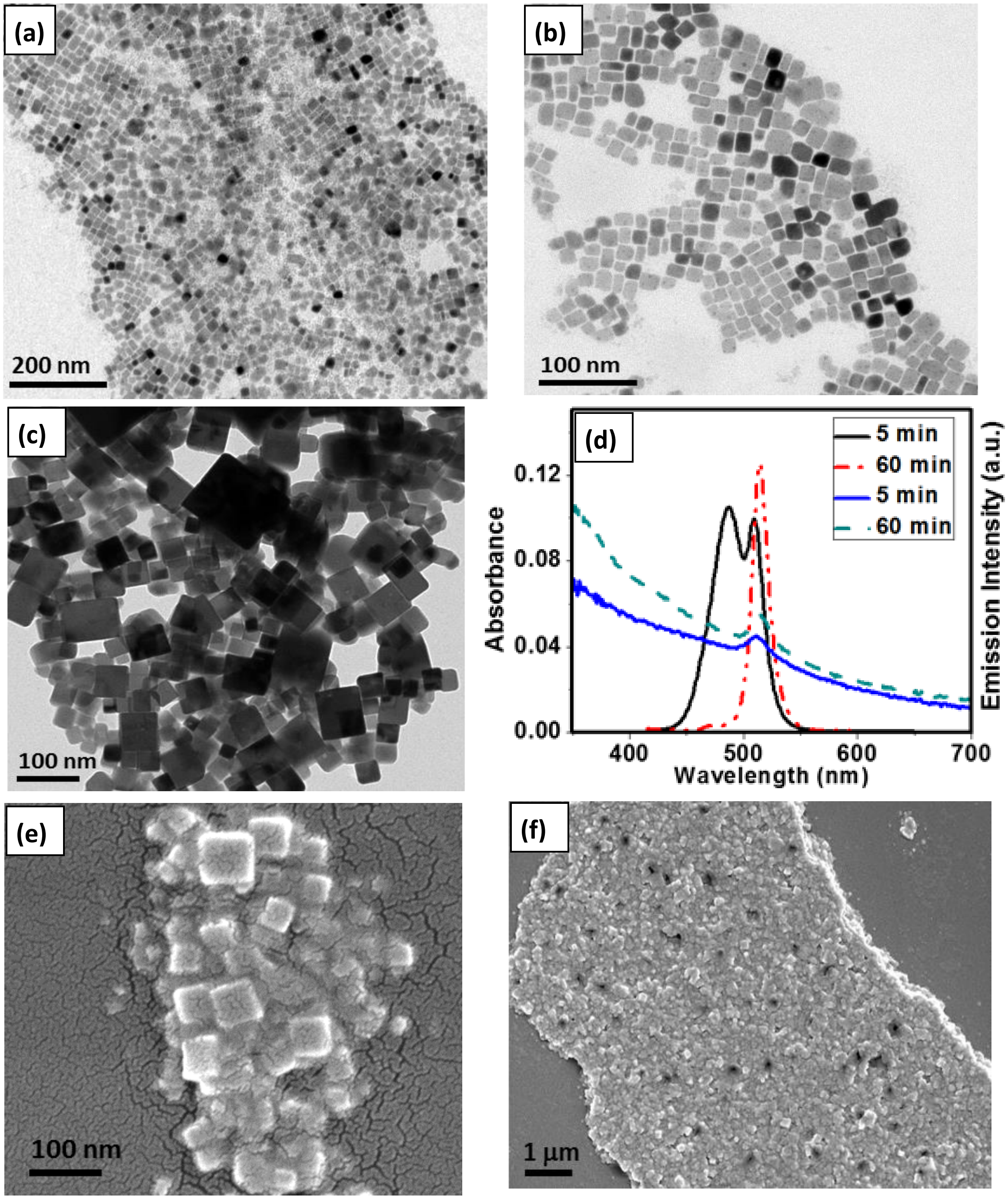

Figure S11. $CsPbBr_3$ nanocubes synthesized in chloroform. TEM images after (a) 1 min, (b) 15 minutes, (c) 60 minutes of the reaction. (d) Absorption and PL spectra at two different times of the reaction. FESEM images after (e) 1 hour and (f) 24 hours of the reaction.

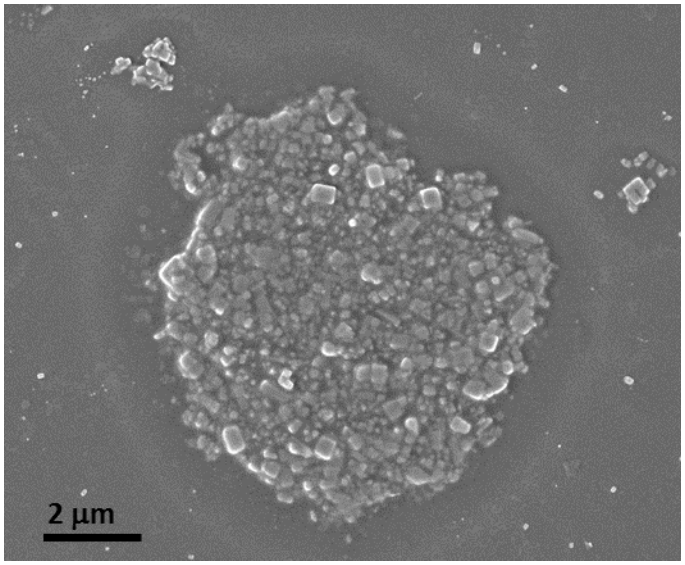

Figure S12. FESEM image of degraded $CsPbBr_3$ nanoparticles formed in n- butanol.

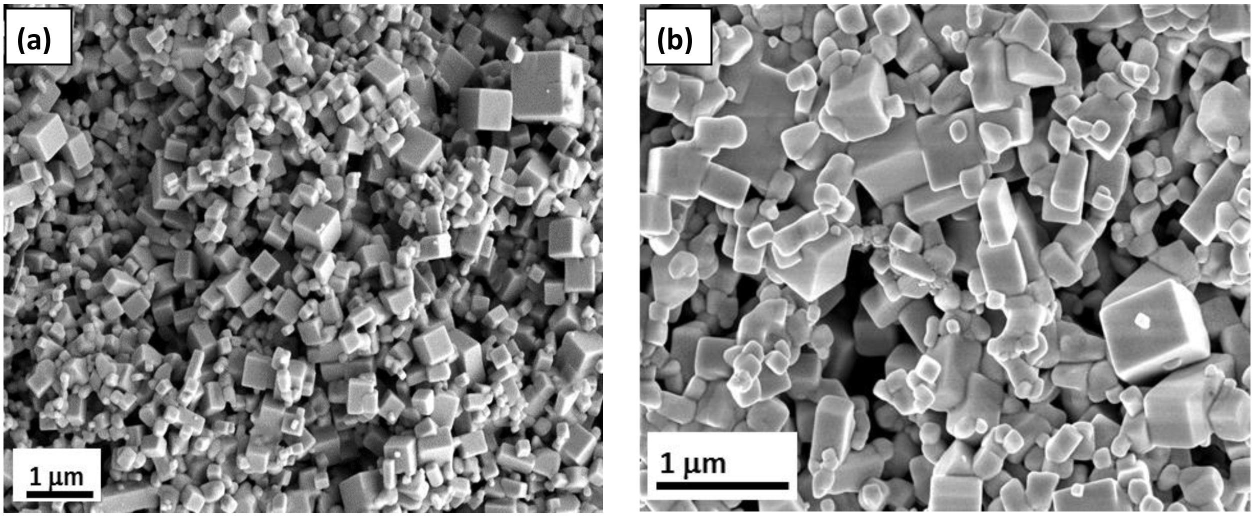

Figure S13. FESEM images of $CsPbBr_3$ nanoparticles formed in ethyl acetate in presence of only oleic acid as capping ligand.

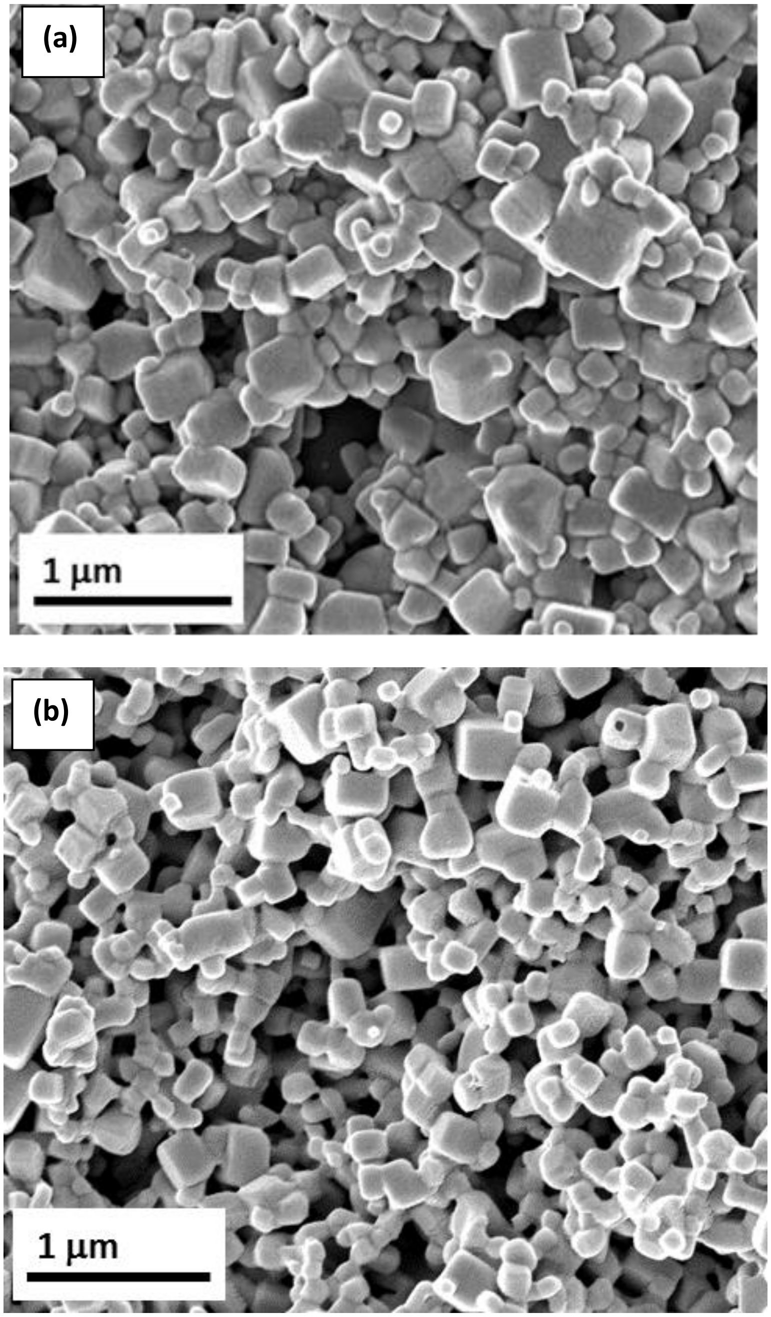

Figure S14. FESEM images of $CsPbBr_3$ nanoparticles formed in (a) ethyl acetate and (b) toluene in absence of any ligand.

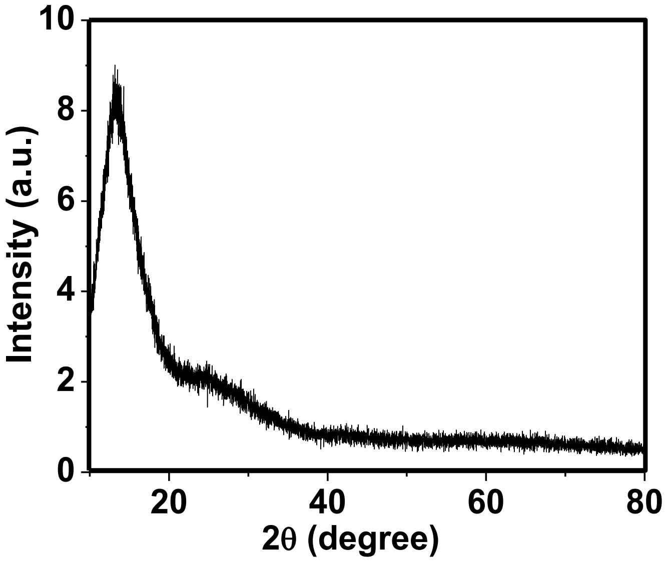

Figure S15. PXRD pattern of a clean coverslip. Presence of the broad peak at ~10-15 degrees is indeed an instrument artifact.

Table S1: Summary of different morphology evolution of $CsPbBr_3$ nanocrystals in 4 mL ethyl acetate at various reaction conditions.

Oleic acid	Oleylamine	Reaction time	Morphology
0 mL	0 mL	1 min	Immediate formation of nonfluorescent yellow precipitate consists of smaller sphere (~100 nm) to larger cubes (~500 nm)
1 mL	0 mL	1 min	Nonfluorescent yellow precipitate. 50-500 nm sized nanocubes and nanobars of sharp edges.
0 mL	20 μL-1 mL	24 hours	No reaction
0.5 mL	100 μL	24 hours	No reaction
0.5 mL	40 μL	1 min	~2.6 nm sized cubo-spherical quantum dots + very few nanoplates
		10 min	Nanoplates of ~60 nm edge length and 5 nm thickness + quantum dots
		4 hours	Quantum dots + nanoplates + nanobars
		10 hours	Very few nanoplates + nanobars (length/width ~100/55 nm)
		25 hours	Nanobars (l/w ~ 140/55 nm)
		40 hours	Nanobars (l/w ~ 140/55 nm)

Table S2: Summary of different morphology evolution of $CsPbBr_3$ nanocrystals in 4 mL toluene at various reaction conditions.

Oleic acid	oleylamine	Reaction time	Morphology
0 mL	0 mL	1 min	Immediate formation of non-fluorescent yellow precipitate. Formed particles are mostly larger nanocubes (120-250 nm).
0 mL	20 μL	24 hours	No reaction
0.5 mL	0 mL	1 min	Immediate formation of non-fluorescent yellow precipitate. Formed particles are of 70-200 nm cubes.
0.5 mL	20-70 μL	1 min	Nanocubes of ~18 nm edge length
0.25 mL	20-70 μL	1 min	Nanocubes of ~12 nm edge length
	70 μL	1 hour	Nanocubes of ~34 nm edge length + small nanocubes
	70 μL	50 hours	Larger nanocubes and few nanorods
	20 μL	1 hours	Nanorods of length ~ 800 nm, diameter ~70 nm + nanocubes
		5 hours	Nanorod length increases (aspect ratio ~70) + nanocubes
		15 hours	Nanorod length increases (aspect ratio ~130) + nanocubes
		25 hours	Nanowires (aspect ratio ~200) + few nanocubes
		40 hours or more	Nanowires (aspect ratio ≥ 200) + very few nanocubes

Table S3: Summary of different morphology evolution of $CsPbBr_3$ nanocrystals in different organic solvents at various reaction conditions.

Solvent	Oleic acid	oleylamine	Reaction time	Morphology
Chloroform 4 mL	0.5 mL	40 μL	1 min	Nanocubes of ~12 nm edge length + some smaller particles emitting at 487 nm.
			15 min	Nanocubes of ~12 nm edge length
			1 hour	Larger nanocubes (≥30 nm) + small nanocubes (12 nm)
			24 hours	Nanoparticles started degrading with complete quenching in the PL.
n-butanol	0.5 mL	40 μL	1 min	Formed nonfluorescent nanoparticles undergo degradation readily and forms a clear solution.
Acetone	0.5 mL	40 μL	10 min	Slow formation of nonfluorescent yellow precipitate at higher precursor concentration
Hexane	0.5 mL	60 μL	1 min	Initially formation of nanocubes of ~12 nm edge length. Later on larger particles. Mostly similar as that obtained in toluene.

Table S4: Summary of the characteristic photoluminescence properties of different $CsPbBr_3$ morphologies.

Morphology	Abs. peak (nm)	PL emission peak (nm)	PL QY (%)	Time resolved PL decay parameters						Amplitude average lifetime <τ> (ns)
				α_1 (ns)	τ_1 (ns)	α_2	τ_2 (ns)	α_3	τ_3	
Quantum dots	437	454	27	0.41	1.27	0.55	4.92	0.04	17.07	3.91
Plates	450	476	19	0.52	2.55	0.41	6.2	0.07	18.10	5.13
Bars	522	522	61	0.57	3.93	0	0	0.43	27.81	14.20
Cubes (~12 nm)	497	510	77	0.73	1.16	0.16	10.95	0.11	79.23	11.31
Cubes (~34 nm)	502	514	54	0.67	1.09	0.20	12.13	0.13	79.87	13.54
Rods	505	516	34	0.65	1.53	0.27	10.82	0.08	62.83	8.94
Wires	511	520	29	0.63	1.54	0.25	9.55	0.12	67.41	11.44
